# *QuickStats:* Percentage* of Persons Who Had a Stomach or Intestinal Illness That Started in the Past 2 Weeks,^†^ by Sex and Age Group — National Health Interview Survey,^§^ 2018

**DOI:** 10.15585/mmwr.mm6904a8

**Published:** 2020-01-31

**Figure:**
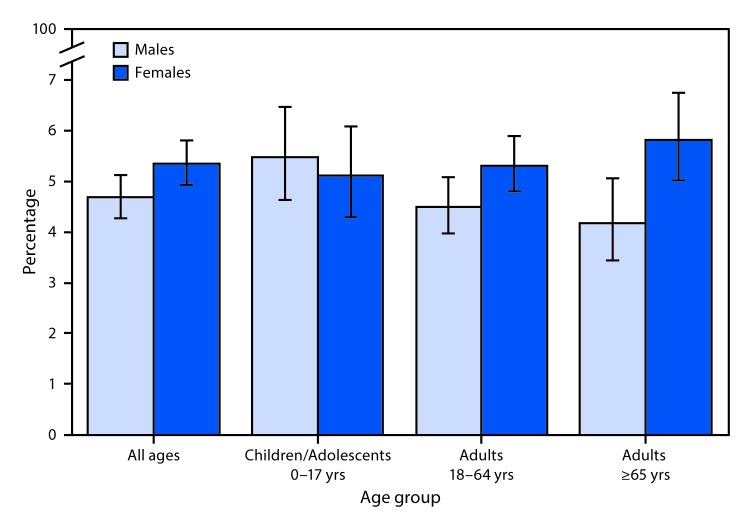
In 2018, 4.7% of males and 5.3% of females had a stomach illness that started in the past 2 weeks. Among children and adolescents aged 0–17 years, no difference was observed in the percentage of males and females who had a stomach illness that started in the past 2 weeks. However, among adults, women were more likely to have had a stomach illness than men. This held for those aged 18–64 years (5.3% of women compared with 4.5% of men) and those aged ≥65 years (5.8% versus 4.2%).

**Source:** National Health Interview Survey, 2018 data. https://www.cdc.gov/nchs/nhis.htm.

